# Novel Podophyllotoxin Derivatives as Partial PPARγ Agonists and their Effects on Insulin Resistance and Type 2 Diabetes

**DOI:** 10.1038/srep37323

**Published:** 2016-11-17

**Authors:** Xiangming Zhang, Huijuan Liu, Bo Sun, Yan Sun, Weilong Zhong, Yanrong Liu, Shuang Chen, Honglei Ling, Lei Zhou, Xiangyan Jing, Yuan Qin, Ting Xiao, Tao Sun, Honggang Zhou, Cheng Yang

**Affiliations:** 1 State Key Laboratory of Medicinal Chemical Biology and College of Pharmacy, Nankai University, Tianjin, China; 2 Tianjin Key Laboratory of Molecular Drug Research, Tianjin International Joint Academy of Biomedicine, Tianjin, China; 3 Department of Obstetrics and Gynecology, General Hospital, Tianjin Medical University, Tianjin, China.

## Abstract

Peroxisome proliferator-activated receptor γ (PPARγ) is recognized as a key regulator of insulin resistance. In this study, we searched for novel PPARγ agonists in a library of structurally diverse organic compounds and determined that podophyllotoxin exhibits partial agonist activity toward PPARγ. Eight novel podophyllotoxin-like derivatives were synthesized and assayed for toxicity and functional activity toward PPARγ to reduce the possible systemic toxic effects of podophyllotoxin and to maintain partial agonist activity toward PPARγ. Cell-based transactivation assays showed that compounds (E)-3-(hydroxy(3,4,5-trimethoxyphenyl)methyl)-4-(4(trifluoromethyl)styryl)dihydrofuran-2(3H)-one (**3a**) and (E)-4-(3-acetylstyryl)-3-(hydroxyl (3,4,5-trimethoxyphenyl)methyl)dihydrofuran-2(3H)-one (**3f**) exhibited partial agonist activity. An experiment using human hepatocarcinoma cells (HepG2) that were induced to become an insulin-resistant model showed that compounds 3a and 3f improved insulin sensitivity and glucose consumption. In addition, compounds 3a and 3f significantly improved hyperglycemia and insulin resistance in high-fat diet-fed streptozotocin (HFD-STZ)-induced type 2 diabetic rats at a dose of 15 mg/kg/day administered orally for 45 days, without significant weight gain. Cell toxicity testing also showed that compounds 3a and 3f exhibited weaker toxicity than pioglitazone. These findings suggested that compounds 3a and 3f improved insulin resistance *in vivo* and *in vitro* and that the compounds exhibited potential for the treatment of type 2 diabetes mellitus.

Peroxisome proliferator-activated receptors (PPARs) are members of the nuclear hormone superfamily of ligand-dependent transcription factors. PPARs control the expression of genes involved in fatty acid and glucose metabolism. They also function as cellular lipid sensors that activate transcription in response to the binding of a cognate ligand, generally fatty acids and their eicosanoid metabolites^1^,^2^,^3^. PPARs include three distinct PPAR subtypes: PPARα, PPARβ, and PPARγ. Among these subtypes, PPARγ serves as the master regulator of adipocyte differentiation and increases the number of small, insulin-sensitive adipocytes^4^. Activation of PPARγ results in insulin-sensitizing antidiabetic effects^5^.

Despite the proven benefits of targeting PPARγ, adverse drug reactions have been reported with PPAR agonists in clinical research^6^. Thiazolidinediones (TZDs) are full PPARγ agonists that exhibit well-established insulin-sensitizing activity^7^. However, as full agonists, they also stimulate adipocyte differentiation *in vitro* and weight gain *in vivo*, which usually worsens the diabetic state. TZD treatment also leads to additional undesirable side effects, including fluid retention, edema, cardiomegaly, anemia, and an increased incidence of bone fractures^8^,^9^. Therefore, there is a need to develop safe and efficacious PPARγ agonists for antidiabetic therapy.

PPARγ agonists exhibit different physical interactions with the receptor and are classified as either full or partial agonists based on their superior efficacy in cell-based transactivation assays^10^. Several studies have shown that partial PPARγ agonists are safer than full PPARγ agonists^1^. Consequently, the ability to retain the favorable glucose-lowering effects of partial PPARγ agonists while avoiding their undesirable side effects presents a major challenge for the development of novel and safer PPARγ-based antidiabetic compounds.

In the search for novel PPARγ agonists, we screened a library of natural products and identified podophyllotoxin (Fig. 1), which is extracted from plants of the genus *Podophyllum*, and found that it exhibits partial activation of PPARγ and the capacity to reverse insulin resistance. However, the application of podophyllotoxin in human clinical trials failed because of its unacceptable gastrointestinal toxic side effects^11^. Chemical modification of podophyllotoxin has led to the derivation of 3-(hydroxy(3,4,5-trimethoxyphenyl)methyl)-4-vinyldihydrofuran-2(3H)-one (PODO-1), which is a nontoxic lead compound for novel PPARγ agonists. We designed and synthesized eight novel PODO-1 derivatives, which were evaluated by various biochemical assays. Among these derivatives, we identified two novel non-TZD derivatives as PPARγ partial agonists. These derivatives are compounds 3a and 3f, chemically known as (E)-3-(hydroxy(3,4,5-trimethoxyphenyl)methyl)-4-(4-(trifluoromethyl)styryl)dihydrofuran-2(3H)-one and (E)-4-(3-acetylstyryl)-3-(hydroxyl (3,4,5-trimethoxyphenyl) methyl) dihydrofuran-2(3H)-one, respectively. These PPARγ partial agonists exhibit lower PPARγ agonistic activity than pioglitazone. The effects of compounds 3a and 3f on hyperglycemia and insulin resistance were also assessed in high-fat-diet-fed streptozotocin HFD-STZ-induced type 2 diabetic rats. Surflex-Dock was used to determine the binding modes of PPARγ and compounds 3a and 3f.

## Results

### Chemistry

**Figure 2:**
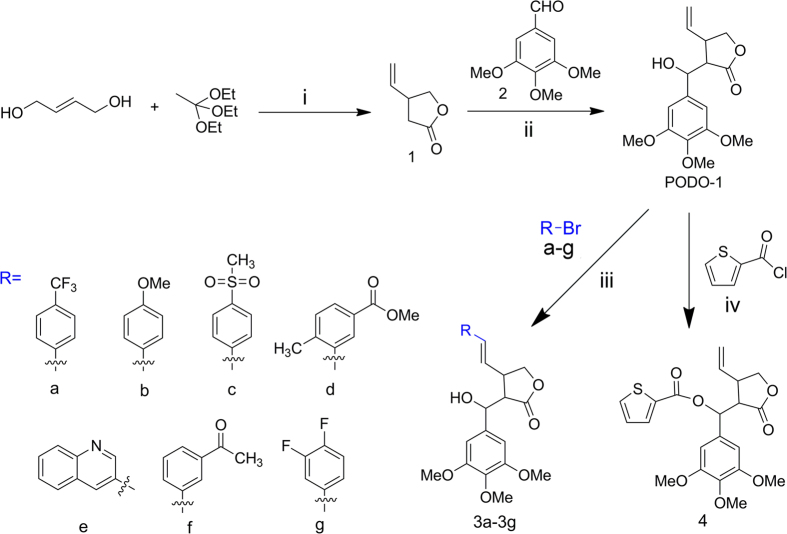
Synthesis of PODO-1 and the PODO-1-derivatives, compounds 3a–3 g and compound 4. i: Hydroquinone, 120 °C–150 °C, atmospheric distillation; ii: diisopropylamine, n-butyllithium, dry THF, 0 °C–−78 °C; iii: Pd(OAc)_2_, PPh3, NEt3, dry DMF, 80 °C; iv: DMAP, NEt_3_, 0 °C–r.t.

PODO-1 was prepared in accordance with previously reported procedures^12^. Compounds 3a–3 g were synthesized using the HECK reaction of PODO-1 with a–g (Fig. 2). The compound PODO-1 was converted to (2-oxo-4-vinyltetrahydrofuran-3-yl) (3,4,5-trimethoxyphenyl)methyl thiophene-2-carboxylate (compound 4) by a reaction with thiophene-2-carbonyl chloride (Fig. 2).

### Transactivation induced by compounds 3a and 3f as selective PPARγ agonists

**Figure 3:**
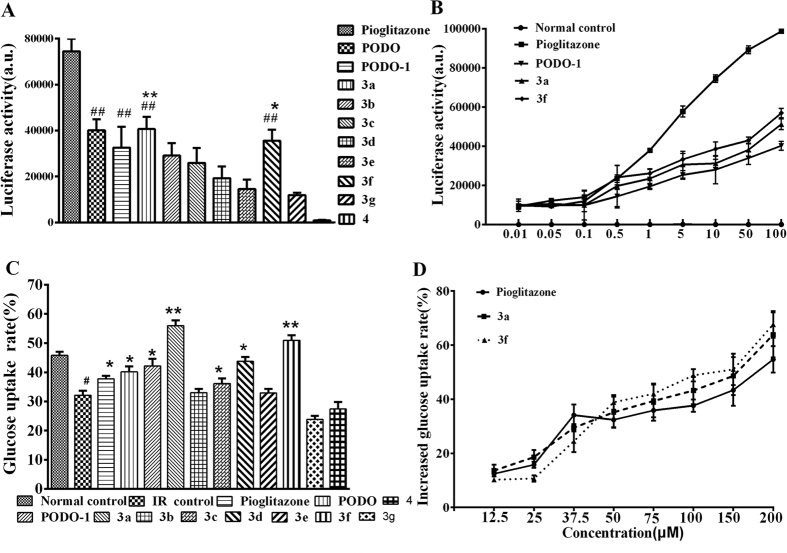
(**A**) PPARγ transactivation assay with 10 μM doses of compounds 3a–3 g, 4, PODO-1, and pioglitazone. *P < 0.05 and **P < 0.01 versus the PODO-1 group; ^##^P < 0.001 versus the pioglitazone group. (**B**) Representative concentration-response curves of reporter gene transactivation by compound 3a, compound 3f, and PODO-1 compared with pioglitazone. (**C**) Insulin-resistant cells were incubated with 20 μM doses of the PODO-1 derivatives and pioglitazone. *P < 0.05 and **P < 0.01 versus the insulin-resistant control; ^#^P < 0.05 versus the normal control. (**D**) Comparative assessment of the glucose uptake potential of compound 3a, compound 3f, and pioglitazone in the insulin-resistant model of HepG2 cells. The standard error bars represent 3 independent experiments, with each experiment performed in triplicate.

The functional potency of compounds 3a and 3f as PPARγ agonists were evaluated using a transient transfection assay in HEK-293T cells. When incubated with HEK-293T cells co-transfected with PPARγ-LBD, a Gal4 chimeric expression vector, and a GAL4-responsive reporter gene plasmid, compounds 3a and 3f induced transactivation in a concentration-dependent manner, with a maximum activation equal to 50% of pioglitazone, indicating that 3a and 3f were partial agonists (Fig. 3A,B).

### Glucose uptake potential of compounds 3a–3f and compound 4 in the dexamethasone(DEX)-induced insulin-resistant model

The effects of compounds 3a-3f and 4 and pioglitazone on insulin resistance were evaluated and compared using the DEX-induced insulin resistant model^13^. Compounds 3a and 3f effectively reversed DEX-induced inhibition of glucose uptake (Fig. 3C). Compounds 3a and 3f maximally increased glucose uptake to approximately 65.13% and 62.25%, respectively, at 200 μM in the DEX-induced insulin-resistant model. The ability of 50–200 μM compounds 3a and 3f to reverse insulin resistance was markedly higher than pioglitazone. In addition, compounds 3a and 3f effectively reversed the DEX-induced inhibition of glucose uptake in a dose-dependent manner (Fig. 3D).

### Cytotoxicity of compound 3a, compound 3f, and pioglitazone in HEK-293, HepG2, NIH-3T3, and Madin-Darby canine kidney (MDCK) cells

**Figure 4:**
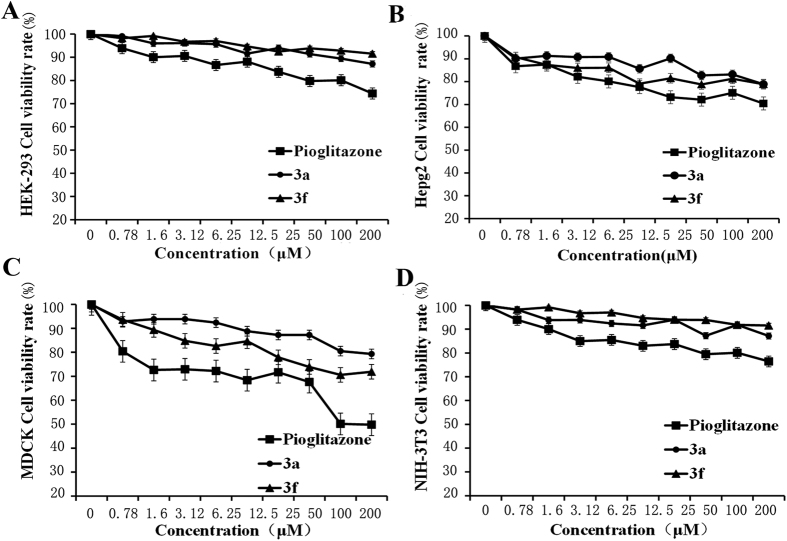
Cytotoxicity of compound 3a, compound 3f, and pioglitazone. Cytotoxic effects of compound 3a, compound 3f, and pioglitazone on (**A**) HEK-293, (**B**) HepG2, (**C**) MDCK, and (**D**) NIH-3T3 cells. The standard error bars represent 3 independent experiments, with each experiment performed in triplicate.

The cytotoxic effects of compound 3a, compound 3f, and pioglitazone on HEK-293, HepG2, NIH-3T3, and MDCK cells were evaluated via a cytotoxicity assay (Fig. 4). Compounds 3a and 3f showed negligible effects on cell viability, whereas pioglitazone exerted approximately 20% to 50% toxicity at its highest concentration. This result showed that compounds 3a and 3f exhibited lower cytotoxicity than pioglitazone.

### Hypoglycemic effect of compounds 3a and 3f on HFD-STZ-induced type 2 diabetic rats

**Figure 5:**
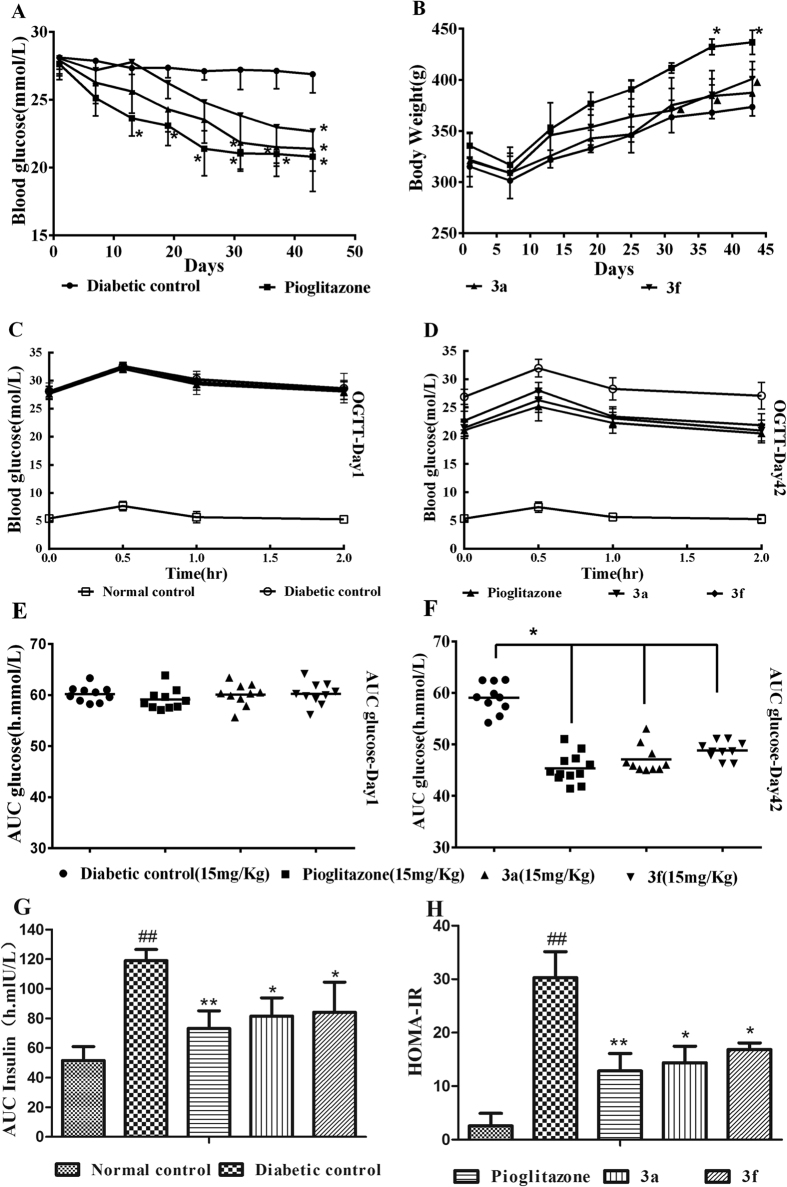
(**A**,**B**) Effects of compound 3a, compound 3f, and pioglitazone on the blood glucose levels and body weights in HFD-STZ-induced diabetic rats. (**C**–**F**) Effects of compounds 3a and 3f on the oral glucose tolerance test in Wistar albino rats. (**C**) Blood glucose levels on Day 1. (**D**) Blood glucose levels on Day 42. (**E**) AUC for glucose on Day 1. (**F**) AUC for glucose on Day 42. (**G**) AUC for insulin during OGTT on Day 42. (**H**) Homeostasis model assessment of insulin resistance (HOMA-IR) index on Day 45. The data are presented as the means ± SD; ^*^P < 0.05 versus the diabetic control, ^**^P < 0.01 versus the diabetic control, ^##^P < 0.01 versus the normal control, ^**^▴^**^P < 0.05 versus pioglitazone.

After administration for 42 days, the fasting serum glucose (FSG) levels of diabetic rats treated with compounds 3a, 3f, and pioglitazone were reduced by 23.3%, 19.18%, and 24.74%, respectively, compared with the FSG levels of the diabetic control rats. Compound 3a produced a significant decrease in the FSG levels from Day 30 to Day 42 (Fig. 5A). Pioglitazone produced a significant increase in body weight, whereas the rats treated with compounds 3a and 3f showed no remarkable change in body weight (Fig. 5B). Oral glucose tolerance tests were performed in HFD-STZ-induced type 2 diabetic rats on Days 1 and 42 of the treatment period. On Day 1, the compound 3a, compound 3f, and pioglitazone treatments did not produce a significant change in glucose levels (Fig. 5C). On Day 42 after chronic treatment, the rats that were administered compounds 3a and 3f (15 mg/kg, orally administered) exhibited reduced glucose levels when challenged with an oral bolus of glucose (indicating increased tolerance to glucose) compared with those of the diabetic control rats (Fig. 5D). The results indicated that compounds 3a and 3f improved the impaired glucose tolerance of type 2 diabetic rats (Fig. 5E,F). The area under the curve of insulin during OGTT (Fig. 5G) showed that compounds 3a and 3f improved the abnormal increased insulin secretion in type 2 diabetic rats. The homeostasis model assessment of insulin resistance (HOMA-IR) index (Fig. 5H) indicated that 3a and 3f effectively decreased insulin resistance in type 2 diabetic rats.

As shown in Table 1, the HFD-STZ-induced diabetic rats had higher serum total cholesterol (TC), triglyceride (TG), and low-density lipoprotein cholesterol (LDL-c) levels and significantly reduced high-density lipoprotein cholesterol (HDL-c) levels compared with those of the normal control rats. The rats that were treated with compounds 3a and pioglitazone had significantly lower serum free fatty acid (FFA), TG, and TC levels compared with those of the diabetic control rats. In addition, the rats that were treated with compound 3a, compound 3f, and pioglitazone had significantly lower LDL-c levels, but significantly higher HDL-c levels compared with those of the diabetic controls.

**Figure 6:**
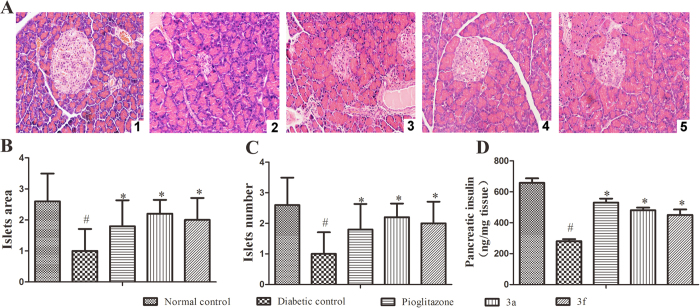
Histopathological changes in the pancreatic islets of the rats in the experimental groups. (**A1**) Pancreatic islet architecture of the normal control rats (H&E, 200× magnification). (**A2**) HFD-STZ-induced diabetic control rats show pancreatic islet damage (H&E, 200×). (**A3**) At 15 mg/kg/day, pioglitazone reversed the pancreatic islet damage (H&E, 200×). (**A4**) At 15 mg/kg/day, compound 3a reversed the pancreatic islet damage (H&E, 200×). (**A5**) At 15 mg/kg/day, compound 3f reversed the pancreatic islet damage (H&E, 200×). (**B**) Analysis of the islet area in the experimental groups. (**C**) Analysis of the islet number in the experimental groups. (**D**) Analysis of the pancreatic insulin levels in the experimental groups. All values are expressed as the means ± SD. Significance was determined by one-way analysis of variance, followed by the Bonferroni post hoc test, ^#^P < 0.05 versus the normal controls; *P < 0.05 versus the diabetic controls.

The histopathological changes in pancreatic islets are illustrated in Fig. 6A,B and C show the area and number of islets in the pancreas. Neither β-cell damage nor inflammatory changes were observed in the normal architecture of pancreatic islets (Fig. 6A1). However, in the HFD-STZ-induced diabetic rats, the β-cells appear to exhibit marked injury and the area of pancreatic islets was markedly reduced (Fig. 6A2). The damage to the pancreatic islets and β-cells in the diabetic rats was repaired using compound 3a, compound 3f, and pioglitazone, as indicated by their protective effects on β-cell damage (Fig. 6A3–A5). Compound 3a and 3f also increased the pancreatic insulin levels in the HFD-STZ-induced diabetic rats (Fig. 6D).

### Molecular docking study of compounds 3a and 3f with human PPARγ

**Figure 7:**
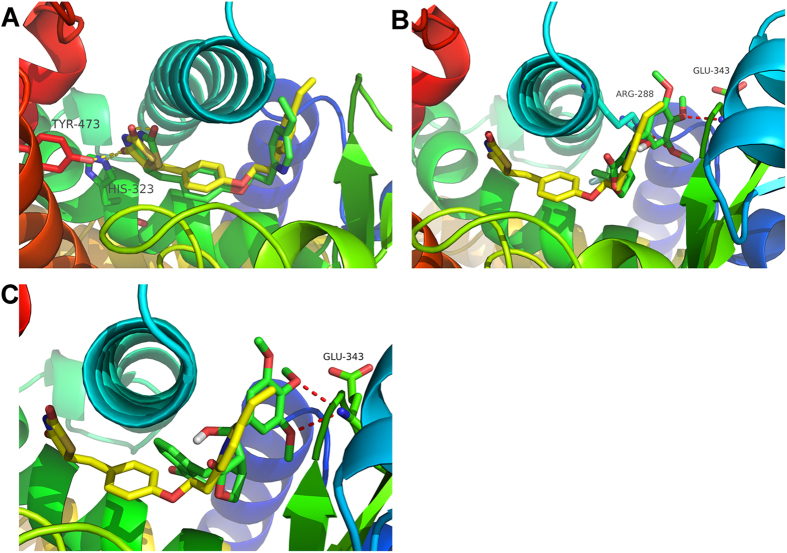
(**A**) Optimal docking conformer of pioglitazone (yellow: crystal structure; green: docking conformer). (**B**) Optimal docking conformer of compound 3a (yellow: crystal structure of pioglitazone; green: docking conformer of compound 3a). (**C**) Optimal docking conformer of compound 3f (yellow: crystal structure of pioglitazone; green: docking conformer of compound 3f).

Pioglitazone was first docked into the binding site of the PPARγ receptor using the same docking protocol as the Surflex-Dock method used to validate compounds 3a and 3f in the current study. Figure 7A shows that an optimal docking conformer of pioglitazone (total score = −8.61) was aligned with the crystal structure (RMSD = 1.15 Å). Figure 7B shows an optimal docking conformer (total score = −7.91) of compound 3a. The binding mode of 3a (green) was apparently different from that of pioglitazone (yellow). Figure 7C presents an optimal docking conformer (total score = −7.40) of compound 3f. The binding mode of compound 3f (green) was quite different from that of pioglitazone (yellow). In the case of pioglitazone, the TZD ring was located at the bottom of the binding pocket, with H-bond interactions with TYR-473 and HIS-323, whereas the pyridine group was located at the entrance. However, for compound 3a, the molecular skeleton was embedded at the entrance, with H-bond interactions with ARG-288 and GLU-343. For compound 3f, the molecular skeleton was also embedded at the entrance, with H-bond interactions with GLU-343. Both compounds 3a and 3f had no moieties located at the bottom of the binding pocket.

## Discussion

PPARγ is a ligand-activated transcription factor that may provide a promising therapeutic approach for metabolic syndrome. TZDs have undesired side effects, including weight gain; thus, there is a need to develop novel partial PPARγ agonists that retain effective insulin-sensitizing action while minimizing the potential side effects. In this study, we searched for novel PPARγ agonists in a library of structurally diverse organic compounds using the transcriptional transactivation assay and identified podophyllotoxin as a partial non-TZD PPARγ ligand. However, podophyllotoxin may exert severe systemic toxic effects^14^. We synthesized a series of new derivatives of podophyllotoxin, namely, compounds 3a–3g and compound 4, to avoid the undesired side effects and maintain the PPARγ partial agonist activity. The results of the luciferase assay indicated that compounds 3a and 3f bound to PPARγ with low affinity compared with the well-characterized PPARγ agonist pioglitazone. The partial PPARγ agonist activity of compounds 3a and 3f may provide a distinct advantage because a number of studies have shown that partial PPARγ agonists, including selective PPAR modulators, produce fewer side effects than full agonists^10^,^15^,^16^.

In the current study, an *in vitro* model that imitates insulin resistance was established to screen insulin-sensitive agents^17^,^18^. The model mimicked the exact *in vivo* clinical insulin-resistant conditions, and the model can be reversed by treatment with insulin-sensitizing drugs^17^,^19^. Similar to pioglitazone, compounds 3a and 3f exhibited the potential to reverse DEX-induced insulin resistance, as evidenced by the restoration of glucose uptake. The results of our *in vitro* studies showed that compounds 3a and 3f not only exhibited strong effects on insulin resistance but also weakened toxicity on cells compared with the full PPARγ agonist pioglitazone.

Feeding rats a HFD and treating them with low-dose STZ (40 mg/kg) can simulate type 2 diabetes^20^. In the present study, the HFD-STZ-treated type 2 diabetic rats showed increased serum glucose levels and β-cell dysfunction, as well as reduced body weight. The results of our *in vivo* studies indicated that compounds 3a and 3f reduced the serum glucose levels and improved the impaired glucose tolerance in type 2 diabetes. Glucose clearance was also significantly increased in rats treated with compounds 3a and 3f, and this finding was confirmed by the AUC analysis. Full PPARγ activation is known to increase body weight. Pioglitazone caused an increase in body weight in the HFD-STZ-treated type 2 diabetic rats, whereas rats treated with compounds 3a and 3f showed no significant change in body weight. Our results also showed that compounds 3a and 3f could continue to improve the fasting serum glucose levels and body weights of the model animals over time.

In the HFD-STZ-induced diabetic rats, dyslipidemia was manifested as increased FFA, LDL-c, TG, and TC levels, as well as reduced HDL-c levels. The hypercholesterolemia and hypertriglyceridemia observed in the model resulted from the increased absorption of TGs and TC from the HFD and elevated concentrations of very low-density lipoproteins, which, consequently, increased the LDL-c levels and reduced HDL-c levels^21^,^22^. In the present study, treatment with compounds 3a and 3f significantly reduced the FFA, TG, TC, and LDL-c levels, but increased the HDL-c levels in the HFD-STZ-induced diabetic rats. Compounds 3a and 3f improve dyslipidemia, possibly by inhibiting hepatic TG secretion or increasing peripheral TG clearance, thus enhancing lipid metabolism and reversing insulin resistance.

Interestingly, the in silico results corroborated the experimental data. The docking scores for pioglitazone (−8.61) against the PPARγ target were higher than those for compound 3a (−7.91) and compound 3f (−7.40), confirming that compounds 3a and 3f exhibit partial agonistic action toward PPARγ. Moreover, based on the results of the molecular docking study, we speculated that the partial PPARγ agonists might be located at the entrance of the binding pocket rather than in the entire pocket. As shown in Fig. 6, the hydroxyl group of compounds 3a and 3f is important for H-bond interactions with amino acid residues. The lack of the hydroxyl group led to the loss of partial agonist activity, which could be the reason why compound 4 almost exhibited no agonist activity in the transcriptional transactivation assay.

In summary, this study showed that compounds 3a and 3f were functionally active as selective partial PPARγ agonists, as determined by the transactivation assay. Our results indicated that compounds 3a and 3f can reduce the FSG levels and reverse dyslipidemia and pancreatic damage, without significantly increasing body weight. In addition, compounds 3a and 3f were less toxic *in vivo* and *in vitro* compared with pioglitazone. Finally, compounds 3a and 3f show potential as candidates for the treatment of type 2 diabetes.

## Materials and Methods

### Compounds and Reagents

3-(4,5-dimethylthiazol-2-yl)-2,5-diphenyltetrazolium bromide (MTT) and dimethyl sulfoxide were purchased from Sangon Biotech (Shanghai, China). The glucose assay kit was purchased from Leadman Group Co., Ltd. (Beijing, China). STZ was purchased from Dalian Meilun Biotech Co., Ltd. (Dalian, Liaoning, China). The insulin assay kit was purchased from shanghai Enzyme-linked Biotechnology (Shanghai, China).

### Cells and Animals

Human hepatocarcinoma HepG2, HEK-293T, HEK-293, NIH-3T3, and Madin-Darby canine kidney (MDCK) cells were supplied by KAIJI Company (Nanjing, China). These cells were maintained in Dulbecco’s Modified Eagle’s Medium (DMEM)/high glucose with 10% fetal bovine serum at 37 °C in a humidified 5% CO_2_ environment. Normoglycemic Wistar albino rats were obtained from Vital River Laboratory Animal Technology Co., Ltd. (Beijing, China).

## Methods

### Synthesis of PODO-1 Derivatives

All reagents were of analytical grade and were dried and purified if necessary. High-resolution mass spectra were recorded on a Quattro LCT (time of flight) spectrometer. ^1^H-NMR and ^13^C-NMR spectra were recorded on a Bruker 400 spectrometer. Chemical shifts were reported in parts per million (ppm) relative to either a tetramethylsilane internal standard or solvent signals. The data were reported as follows: chemical shift, multiplicity (s = singlet, d = doublet, m = multiplet), coupling constants, and integration. Thin layer chromatography was conducted using SG SGF254 F254 sheets (Taiyang, Rushan, China), and the spots were visualized using UV at 254 and 365 nm.

### Typical procedure for the synthesis of the PODO-1 analogs (compounds 3a–3g)

Synthesis of compounds 3a–3g: A two-necked round-bottom flask was charged with PODO-1 (0.172g, 0.558 mmol), Pd(OAc)_2_ (6.25 mg, 0.0279 mmol), PPh3 (8.77 mg, 0.03348 mmol), NEt_3_ (0.113g, 1.117 mmol), 30 mL of dry DMF and compounds a–g (1.117 mmol). The mixture was heated to 80 °C (higher temperature led to further decomposition) and stirred for 20 h. After cooling to room temperature, the suspension was filtered over a short pad of silica, washed with an additional 20 mL of CH_2_Cl_2_, and concentrated under reduced pressure. The obtained crude product was purified by flash chromatography (PE:EA = 4:1) to obtain compounds 3a–3g in the solid state. The NMR-H spectrum of compound 3a-3g was supplied in supplementary information Figure S1–S7.

Compound 3a (yield: 86%): ^1^H NMR (400 MHz, CDCl_3_): δ 7.49 (d, *J* = 8.1 Hz, 2H), 7.19 (d, *J* = 8.1 Hz, 2H), 6.16 (d, *J* = 15.8 Hz, 1H), 5.78 (dd, *J* = 15.8, 8.3Hz, 1H), 5.4 (s, 1H), 4.46 (t, *J* = 8.7 Hz, 1H), 3.99 (t, *J* = 8.7 Hz, 1H), 3.81 (s, 6H), 3.58 (s, 3H) 3.25 (d, 1H), 2.89 (dd, 1 H). ^13^C NMR (101 MHz, CDCl_3_): δ 177.10, 153.34, 139.61, 137.27, 136.41, 131.29, 129.62, 126.16, 125.56, 125.52, 102.74, 70.52, 70.31, 60.47, 56.18, 53.41, 38.88. HRMS (m/z): [M + Na]^+^ calcd. for C_20_H_15_C_l2_NO_5_Na, 475.1344; found, 475.1353.

Compound 3b (yield: 63%): ^1^H NMR (400 MHz, CDCl_3_): δ 7.89 (dd, *J* = 3.8, 1.2 Hz, 1H), 7.62 (dd, *J* = 5.0, 1.2 Hz, 1H), 7.15 (dd, *J* = 4.9, 3.8 Hz, 1H), 6.63 (s, 2H), 6.38 (d, *J* = 3.8 Hz, 1H), 5.63–5.51 (m, 1H), 5.06 (t, *J* = 5.3Hz, 1H), 5.03 (s, 1H), 4.43 (t, *J* = 8.7 Hz, 1H), 3.96 (t, *J* = 9.0 Hz, 1H), 3.84 (d, *J* = 2.7 Hz, 6H), 3.83 (s, 3H), 3.39 (p, *J* = 8.6 Hz, 1H), 3.02 (dd, *J* = 9.8, 3.8 Hz, 1H). ^13^C NMR (101 MHz, CDCl_3_): δ 177.43, 159.47, 153.34, 137.39, 136.27, 132.28, 127.20, 124.31, 123.03, 114.03, 102.94, 74.58, 70.89, 60.63, 56.18, 55.30, 53.17, 39.36. HRMS (m/z): [M + Na]^+^ calcd. for C_23_H_26_O_7_Na, 437.1576; found, 437.1580.

Compound 3c (yield: 80%): ^1^H NMR (400 MHz, CDCl_3_): δ 7.82 (d, *J* = 8.4 Hz, 2H), 7.27 (d, *J* = 8.4 Hz, 2H), 6.64 (s, 2H), 6.12 (d, *J* = 15.8 Hz, 1H), 5.81 (dd, *J* = 15.8, 1H), 4.88 (d, *J* = 7.8 Hz, 1H), 4.40 (t, *J* = 8.7 Hz, 1H), 4.01 (t, *J* = 8.7 Hz, 1H), 3.83 (s, 6H), 3.59 (s, 3H), 3.11 (t, *J* = 9.6, 1H), 3.03 (s, 3H), 2.88 (m, 1H). ^13^C NMR (101 MHz, CDCl_3_): δ 177.85, 153.43, 141.38, 139.45, 138.15, 135.21, 131.04, 129.84, 127.80, 126.71, 104.09, 74.39, 70.31, 60.44, 56.25, 52.06, 44.50, 42.44. HRMS (m/z): [M + Na]^+^ calcd. for C_23_H_26_O_8_SNa, 463.1348; found, 463.1420.

Compound 3d (yield: 47%): ^1^H NMR (400 MHz, CDCl_3_): δ 7.80 (s, 1H), 7.75 (dd, *J* = 8.0, 1.5 Hz, 1H), 7.12 (d, *J* = 8.0 Hz, 1H), 6.62 (s, 2H), 6.22 (d, *J* = 15.5 Hz, 1H), 5.73 (dd, *J* = 15.5, 8.6 Hz, 1H), 4.84 (d, *J* = 7.8 Hz, 1H), 4.38 (t, *J* = 8.8 Hz, 1H), 4.22 (s, 1H), 3.99 (t, *J* = 9.5 Hz, 1H), 3.90 (s, 3H), 3.81 (s, 6 H), 3.40(s, 3H), 3.09 (m, 1H), 2.22 (s, 3H). ^13^C NMR (151 MHz, CDCl_3_): δ 178.24, 166.89, 153.35, 140.36, 137.96, 135.31, 135.17, 130.46, 129.44, 128.65, 128.10, 128.06, 126.35, 104.06, 74.43, 70.70, 60.18, 56.13, 52.05, 51.91, 42.93, 19.69. HRMS (m/z): [M + Na]^+^ calcd. for C_23_H_28_O_8_Na, 479.1682; found, 479.1667.

Compound 3e (yield: 58%) ^1^H NMR (400 MHz, CDCl_3_): δ 8.76 (s, 1H), 8.08 (d, *J* = 8.3Hz, 1H), 7.81 (s, 1H), 7.79 (d, 1H), 7.71 (m, 1H), 7.57 (m, 1H), 6.68 (s, 2H), 6.31 (d, *J* = 15.9 Hz, 1H), 5.96 (dd, *J* = 15.9, 8.4 Hz, 1H), 5.45 (d, *J* = 2.5 Hz, 1H), 4.52 (t, *J* = 8.7 Hz, 1H), 4.33 (t, *J* = 6.7 Hz, 1H), 4.08 (t, *J* = 8.7 Hz, 1H), 3.84 (s, 6H), 3.55 (s, 3H), 2.98 (dd, *J* = 10.0, 3.0 Hz, 1H). ^13^C NMR (101 MHz, CDCl_3_): δ 177.07, 153.37, 148.48, 147.26, 137.32, 136.67, 132.48 (s), 129.79, 129.53, 129.31, 129.13, 128.96, 127.78, 127.20, 102.87, 70.60, 70.34, 60.58, 56.22, 53.45, 39.19. HRMS (m/z): [M + Na]^+^ calcd. for C_25_H_25_NO_6_Na, 436.1760; found, 436.1757.

Compound 3f (yield: 61%) ^1^H NMR (400 MHz, CDCl_3_): δ 7.79 (d, *J* = 7.7 Hz, 1H), 7.74 (s, 1H), 7.37 (t, *J* = 7.7 Hz, 1H), 7.30 (s, 1H), 6.64 (s, 2H), 6.21 (d, *J* = 15.8 Hz, 1H), 5.81 (dd, *J* = 15.8, 8.4 Hz, 1H), 5.41 (m, 1H), 4.47 (t, *J* = 8.7 Hz, 1H), 4.01 (t, *J* = 8.7 Hz, 1H), 3.83 (s, 6H), 3.60 (s, 3H), 2.93 (dd, *J* = 10.2, 3.1 Hz, 1H), 2.82 (d, *J* = 5.2 Hz, 1H), 2.61 (s, 3H). ^13^C NMR (101 MHz, CDCl_3_): δ 197.76, 176.99, 153.40, 137.41, 136.72, 136.22, 131.90, 130.50, 128.91, 128.19, 127.79, 125.52, 102.75, 70.60, 60.53, 56.20, 53.22, 39.19, 26.65. HRMS (m/z): [M + Na]^+^ calcd. for C_24_H_26_O_7_Na, 449.1576; found, 449.1594.

Compound 3 g (yield: 49%) ^1^H NMR (400 MHz, CDCl_3_): δ 7.05 (m, 1H), 6.92 (m, 1H), 6.79 (m, 1H), 6.62 (s, 2H), 5.97 (d, *J* = 15.8 Hz, 1H), 5.54 = (dd, *J* = 15.8, 8.3Hz, 1H), 4.85 (d, 1H), 4.38 (t, *J* = 8.7 Hz, 1H), 3.98 (t, *J* = 8.7 Hz, 1H), 3.83 (s, 6H), 3.66 (s, 1H), 3.61 (s, 3H), 3.04 (m, 1H), 2.82 (dd, 1H). ^13^C NMR (101 MHz, CDCl_3_): δ 178.07, 153.41, 151.61, 148.93, 138.16, 135.16, 130.72, 126.68, 122.37, 117.44, 114.31, 104.03, 74.41, 70.48, 60.55, 56.21, 52.00, 42.38. HRMS (m/z): [M + Na]^+^ calcd. for C_22_H_22_F_2_O_6_Na, 443.1282; found, 443.1292.

### Procedure for the synthesis of compound 4

A two-necked round-bottom flask was charged with PODO-1 (150 mg, 0.487 mmol), NEt3 (589.8 mg, 5.84 mmol) and DMAP (5.94 mg, 0.0487 mmol). The mixture was protected under nitrogen atmosphere and cooled to 0 °C. Thiophene-2-carbonyl chloride (142.2 mg, 0.974 mmol) was added dropwise at 0 °C, and the reaction mixture was stirred at room temperature for 5 h. The reaction was quenched by the rapid addition of H_2_O, and the aqueous layer was extracted using ethyl acetate (3 × 10 mL). The combined organic layers were washed with a saturated aqueous citrate solution (20 mL), saturated aqueous NaHCO_3_ (20 mL), and saturated aqueous NaCl (20 mL), dried over Na_2_SO_4_, filtered, and concentrated under reduced pressure. The crude product was purified by flash chromatography (PE: EA = 5:1) to yield compound 4 (166 mg, 0.399 mmol, yield 82%). The NMR-H spectrum of compound 4 was supplied in supplementary information Figure S8.

Compound 4 (yield: 73%): ^1^H NMR (400 MHz, CDCl_3_): δ ppm 7.89 (dd, *J* = 3.8, 1.2 Hz, 1H), 7.62 (dd, *J* = 5.0, 1.2 Hz, 1H), 7.15 (dd, *J* = 4.9, 3.8 Hz, 1H), 6.63 (s, 2H), 6.38 (d, *J* = 3.8 Hz, 1H), 5.63–5.51 (m, 1H), 5.06 (d, *J* = 5.8 Hz, 1H), 5.03 (s, 1H), 4.43 (t, *J* = 8.7 Hz, 1H), 3.96 (t, *J* = 9.0 Hz, 1H), 3.84 (s, 6H), 3.83 (s, 3H), 3.39 (p, *J* = 8.6 Hz, 1H), 3.02 (dd, *J* = 9.8, 3.8 Hz, 1H). ^13^C NMR (101 MHz, CDCl_3_): δ ppm = 174.69, 160.47, 153.43, 138.04, 135.26, 134.27, 133.00, 132.76, 132.72, 128.10, 118.08, 103.46, 73.36, 70.26, 60.85, 56.17, 51.19, 41.36. HRMS (m/z): [M + Na]^+^ calcd. for C_21_H_22_O_7_SNa, 441.0984; found, 441.0977.

### Transcriptional transactivation assay

HEK-293T cells were grown in DMEM with 10% fetal bovine serum at 37 °C in 5% CO_2_ and then seeded in 96-well plates at a density of 2 × 10^4^ cells per well. The ligand binding domains of PPARγ were generated by PCR amplification using Pfu polymerase and gene-specific primers flanked with sites for the restriction enzymes BamHI and XbaI. Approximately 204–505 residues of human PPARγ-LBD were cloned into the pGal4-DBD (pBIND) to generate fusion proteins with the DNA-binding domain of GAL4. The pGal4-DBD (pBIND)-PPARγ-ligand-binding domain (LBD) fusion vector (0.05 mg/well) and pG5luc vector (0.15 mg/well) were cotransfected into HEK-293T cells for 6 h to measure the transcriptional transactivation activities of the compounds using the luciferase reporter assay^23^. The Lipofectamine 2000 transfection reagent was used for transfection. The cells were subsequently treated with various PODO-1 derivatives or pioglitazone for 24 h. Firefly luciferase activity was measured using the ONE-Glo^TM^ Luciferase Assay System (Promega Corp., Madison, WI).

### Effect of PODO-1 and its derivatives on glucose uptake in insulin-resistant HepG2 cells

HepG2 cells were seeded in 96-well plates at a density of 1 × 10^4^ cells per well and then incubated in serum-free DMEM/high glucose overnight. The cells were subsequently induced with 100 nM DEX for 24 h. After 24 h, the cells were washed 3 times with phosphate-buffered saline and stimulated with 1 nM insulin for 24 h. A glucose assay kit was used to detect glucose consumption by measuring the glucose levels in the media via the glucose oxidase-peroxidase method. We then calculated the glucose consumption. The normal control cells that were induced to be insulin-resistant were treated with compounds 3a–3g, compound 4, and pioglitazone for 24 h to evaluate the effects of the PODO-1 derivatives and pioglitazone on glucose uptake.

### Cytotoxicity assessment

The cytotoxic effects of compounds 3a and 3f and pioglitazone on HepG2, NIH-3T3, HEK-293, and MDCK cells were evaluated using the MTT reagent. The assay was performed 24 h after treatment with compounds 3a and 3f and pioglitazone. The formazan concentration, which is directly proportional to cell viability, was measured at 492 nm.

### Animals and drug treatments

The Institutional Animal Ethical Committee of Nankai University approved the experimental protocol, and all procedures involving animals were performed in accordance with the Guidelines for the Care and Use of Laboratory Animals from the National Institutes of Health. With the exception of the rats in the normal control group, all rats (6 weeks old, 180 g–200 g) were fed a high-fat diet (HFD) consisting of 20% glucose, 10% egg yolk powder, 10% lard, 0.2% bile salts, 1.5% cholesterol, and 58.3% normal commercial pellet diet. After 10 days of feeding on the HFD, the rats were fasted overnight for 12 h and then given a single injection of a freshly prepared solution of STZ (40 mg/kg) in citrate-phosphate buffer (0.1 M, pH 4.2)^24^. Hyperglycemia was assessed by measuring the fasting serum glucose (FSG) levels in the rats 72 h after the STZ administration. Rats with FSG levels higher than 13.89 mmol/L were selected for the subsequent experiments. The rats were randomly divided into 5 groups of 10 animals each: the normal control group (rats without any treatment); the diabetic control group (diabetic rats treated with vehicle); the positive control group (diabetic rats treated with 15 mg/kg per day pioglitazone by oral gavage); the group treated with compound 3a (diabetic rats treated with 15 mg/kg per day compound 3a by oral gavage); and the group treated with compound 3f (diabetic rats treated with 15 mg/kg per day compound 3f by oral gavage). The rats were treated for 45 days. An oral glucose tolerance test (OGTT) was performed on Days 1 and 42. During the OGTT assay, an oral dose of vehicle or the compounds was administered to the 12 h-fasted rats. The rats then received an oral administration of a glucose bolus (2 g/kg) by gavage after their baseline blood glucose levels were measured. The blood glucose levels and the insulin levels were then measured at 0, 30, 60, and 120 min.

On Day 45, a blood sample was withdrawn from the heart. After separation by centrifugation at 3,000 g for 10 min, the serum was separated and analyzed to determine the free fatty acid (FFA), high-density lipoprotein cholesterol (HDL-c), low-density lipoprotein cholesterol (LDL-c) triglyceride (TG), and total cholesterol (TC) levels. The HOMA-IR index was calculated using the following equation: fasting insulin (mU/L) × fasting glucose (mmol/L)/22.5.

The animals were dissected, the spleen side of the pancreas was used to measure insulin content following extraction with the acid ethanol method, and the remainder was subjected to histopathological studies^25^. Formalin-fixed pancreatic tissue was cut into 4-mm-thick sections and then stained with H&E^24^. The stained histological sections were randomly captured with a light microscope (Nikon, Tokyo, Japan).

### Molecular docking

Surflex-Dock was employed using MOE 2013 to determine the binding mode of PPARγ and compounds 3a and 3f. Surflex-Dock is a fully automatic flexible molecular docking algorithm based on molecular similarity and an empirical-based scoring function^26^. The docking score is expressed in -lg(Kd) unit, which consists of hydrophobic, polar, electrostatic, repulsive, entropic, and solvation terms. The crystal structure of PPARγ was derived from the PDB database (ID: 2XKW). The co-crystal with the pioglitazone ligand was used to generate a protocol with a threshold and bloat of 0.30 and 2.0 Å, respectively. Prior to docking, compounds 3a and 3f were optimized using Tripos force field and MMFF94 partial charges. The numbers of additional starting conformations and resulting docking poses were set to 5 and 20, respectively.

### Statistical analysis

The data in the tables and figures are expressed as the means ±SD. All data were analyzed using one-way ANOVA, followed by Bonferroni post hoc test (SPSS version 17.0; SPSS Inc., Chicago, IL), and statistical significance was defined as P < 0.05.

## Additional Information

**How to cite this article**: Zhang, X. *et al.* Novel Podophyllotoxin Derivatives as Partial PPARγ Agonists and their Effects on Insulin Resistance and Type 2 Diabetes. *Sci. Rep.* **6**, 37323; doi: 10.1038/srep37323 (2016).

**Publisher’s note**: Springer Nature remains neutral with regard to jurisdictional claims in published maps and institutional affiliations.

## Supplementary Material

Supplementary Information

## Figures and Tables

**Figure 1:**
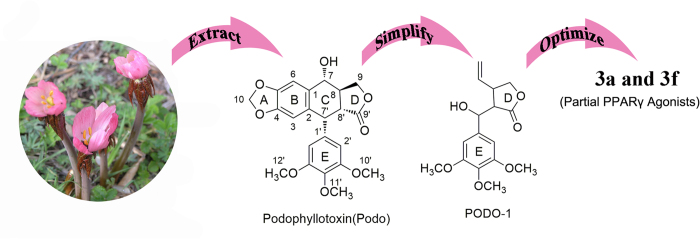
Process for discovering partial PPARγ agonists: compounds 3a and 3f.

**Table 1:** Effects of compounds 3a and 3f on the biochemical parameters of normal and diabetic rats.

Biochemical Parameter	Normal Control	Diabetic Control	Pioglitazone 15 mg/kg	Compound 3a 15 mg/kg	Compound 3f 15 mg/kg
FFA (mmol/L)	0.16 ± 0.03	0.21 ± 0.01^a^	0.15 ± 0.01^b^	0.17 ± 0.03^b^	0.16 ± 0.02^b^
HDL-c (mmol/L)	0.83 ± 0.01	0.61 ± 0.16^a^	0.72 ± 0.15^b^	0.64 ± 0.09	0.66 ± 0.12^b^
LDL-c (mmol/L)	0.24 ± 0.03	2.92 ± 1.13^a^	1.60 ± 0.14^b^	1.35 ± 0.25^b^	1.17 ± 0.43^b^
TC (mmol/L)	1.53 ± 0.18	8.91 ± 3.44^a^	5.20 ± 1.25^b^	4.71 ± 0.51^b^	3.81 ± 1.23^b^
TG (mmol/L)	0.71 ± 0.05	1.18 ± 0.21^a^	0.66 ± 0.23^b^	0.68 ± 0.21^b^	0.49 ± 0.23^b^

All values are expressed as the means ± SD. The level of significance was determined by one-way ANOVA, followed by the Bonferroni post hoc test. ^a^P < 0.01 versus the normal control. ^b^P < 0.05 versus the diabetic control.
