# Supplementary material for: Novel Podophyllotoxin Derivatives as Partial PPARγ Agonists and their Effects on Insulin Resistance and Type 2 Diabetes
Source: Sci Rep. 2016 Nov 17;6:37323. doi: 10.1038/srep37323 (PMC5112511; doi:10.1038/srep37323)

**Novel Podophyllotoxin Derivatives as Partial PPARγ Agonists and their Effects on Insulin Resistance and Type 2 Diabetes**

Xiangming Zhang^#1^, Huijuan Liu^#2^, Bo Sun^#2^, Yan Sun^3^, Weilong Zhong ^1^, Yanrong Liu ^2^, Shuang Chen ^2^, Honglei Ling ^2^, Lei Zhou ^2^, Xiangyan Jing ^2^, Yuan Qin ^1^, Ting Xiao ^1^, Tao Sun *^1,2^, Honggang Zhou *^1,2^, Cheng Yang*^1,2^

^1^State Key Laboratory of Medicinal Chemical Biology and College of Pharmacy, Nankai University, Tianjin, China. ^2^Tianjin Key Laboratory of Molecular Drug Research, Tianjin International Joint Academy of Biomedicine, Tianjin, China. ^3^Department of  Obstetrics and Gynecology, General Hospital, Tianjin Medical University.^#^These authors contribute equally to this work. *Correspondence and requests for materials should be addressed to: Cheng Yang ([cyang66_2001@yahoo.com](mailto:cyang66_2001@yahoo.com)) or Honggang Zhou ([honggang.zhou@vip.126.com](mailto:honggang.zhou@vip.126.com)) or Tao Sun ([sunrockmia@hotmial.com](mailto:sunrockmia@hotmial.com)) .

**Supporting Information**

Figure S1 NMR-H spectrum of compound 3a (CDCl3, 400 MHz)


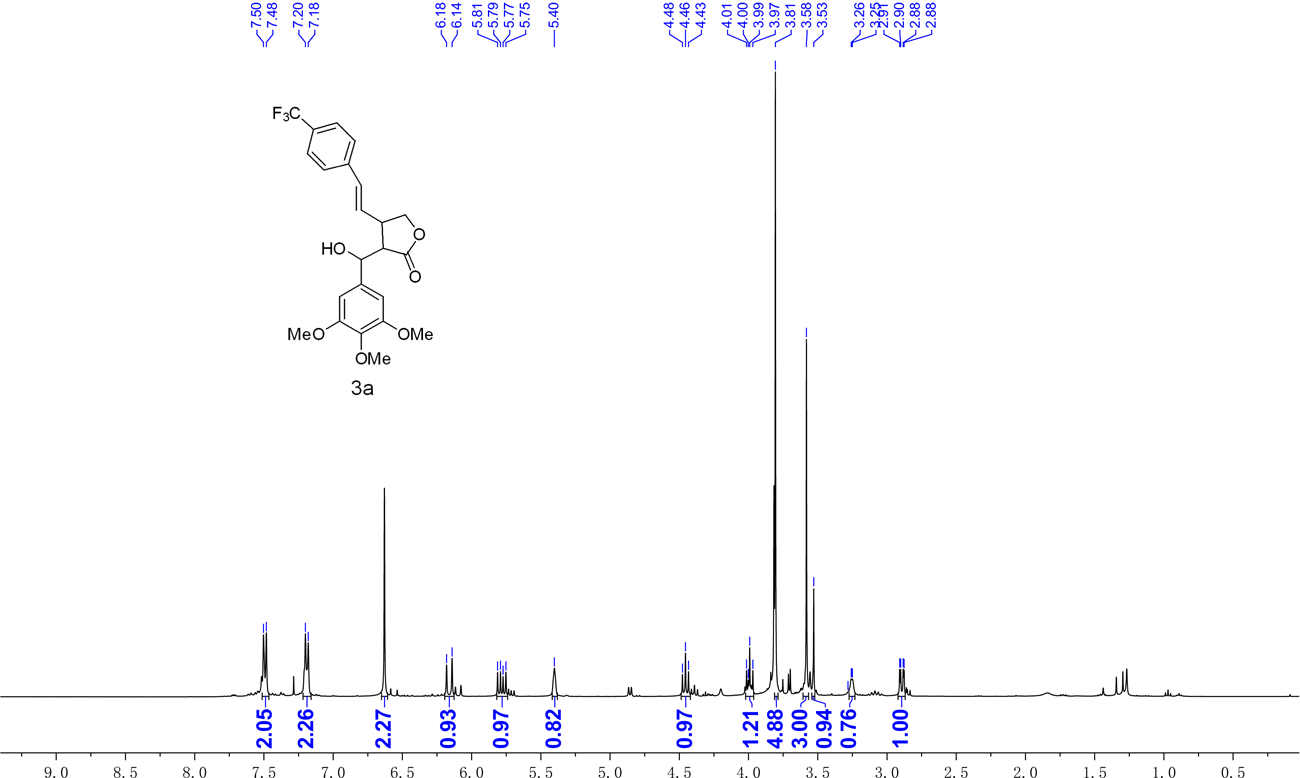


Figure S2 NMR-H spectrum of compound 3b (CDCl3,400MHz)
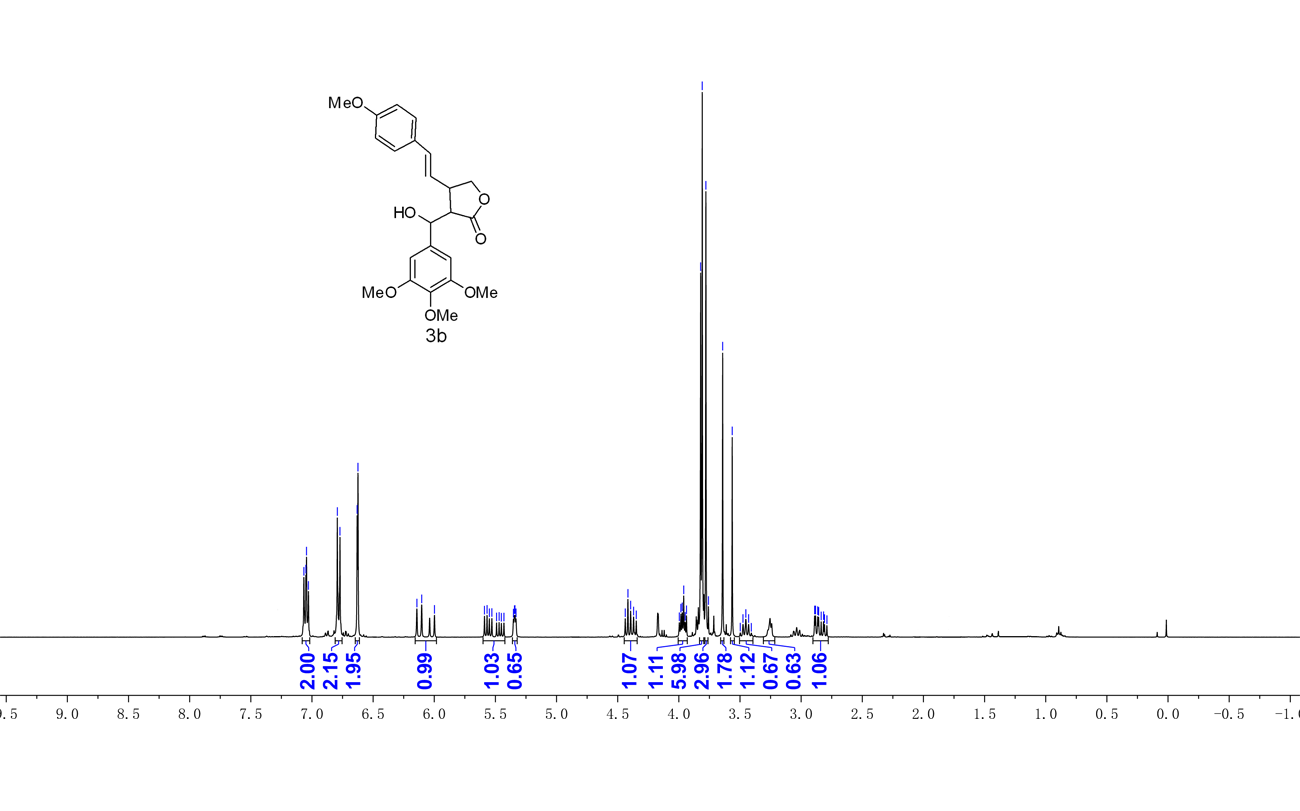


Figure S3 NMR-H spectrum of compound 3c (CDCl3,400MHz)


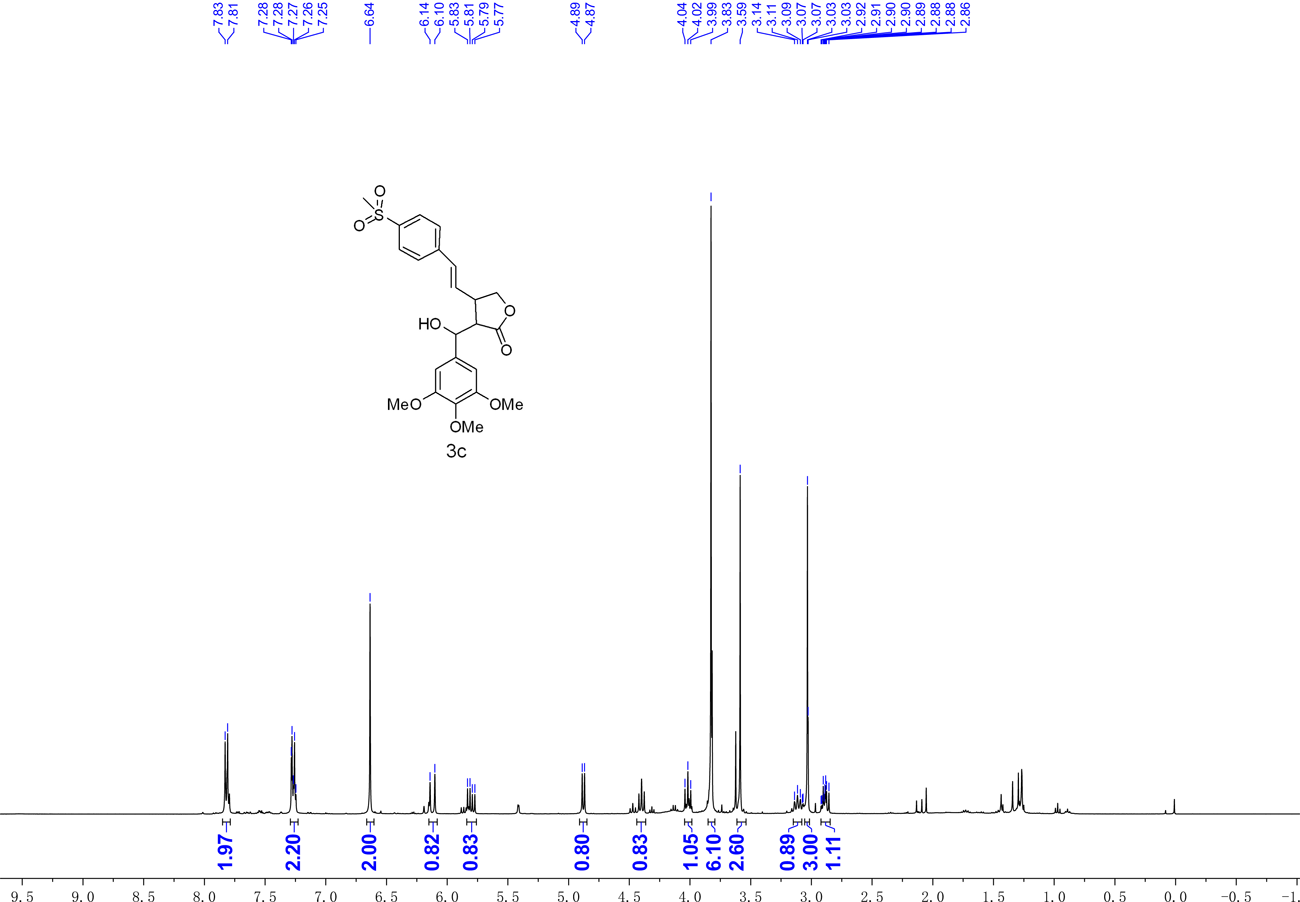


Figure S4 NMR-H spectrum of compound 3d (CDCl3,400MHz)


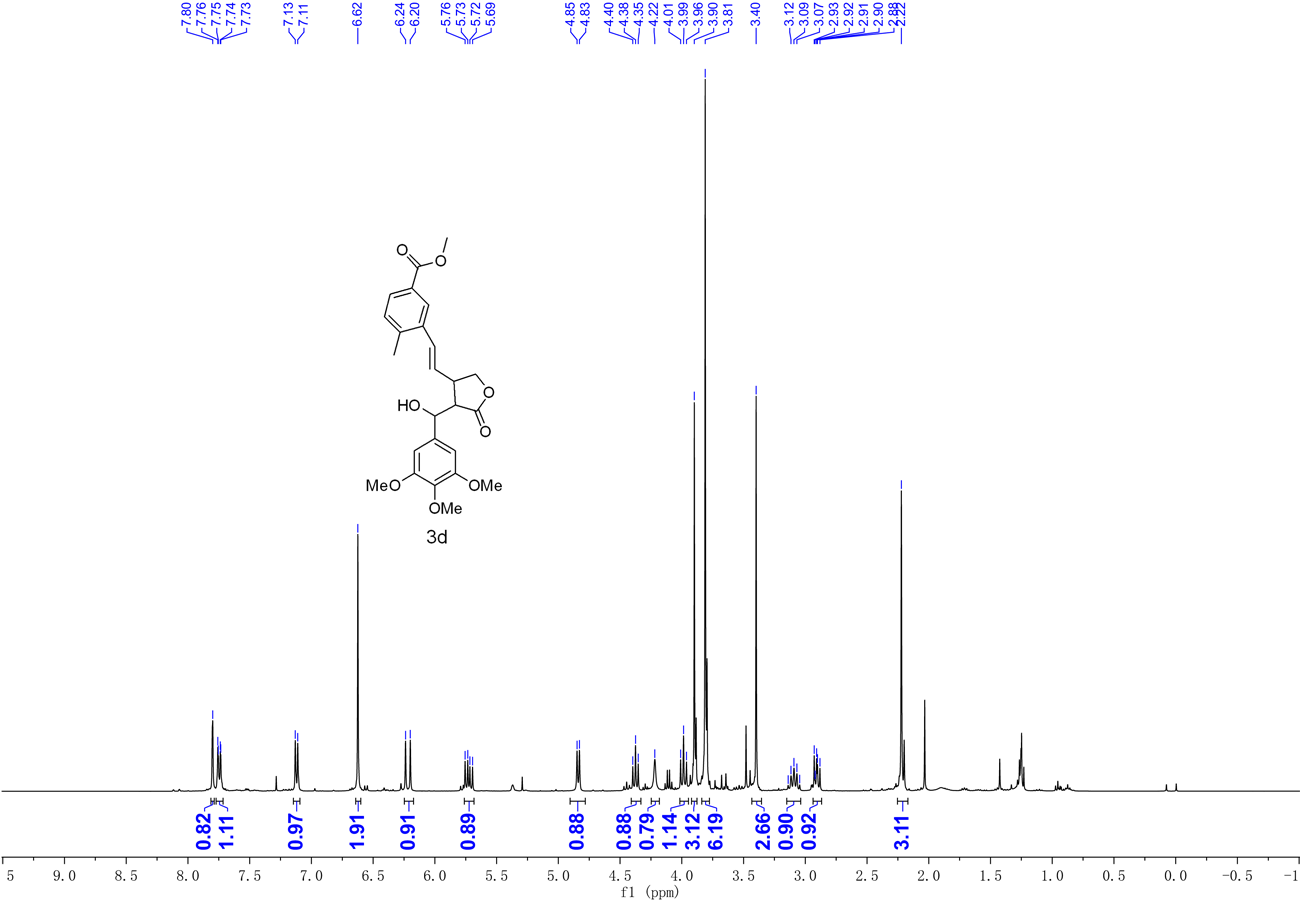


Figure S5 NMR-H spectrum of compound 3e (CDCl3,400MHz)


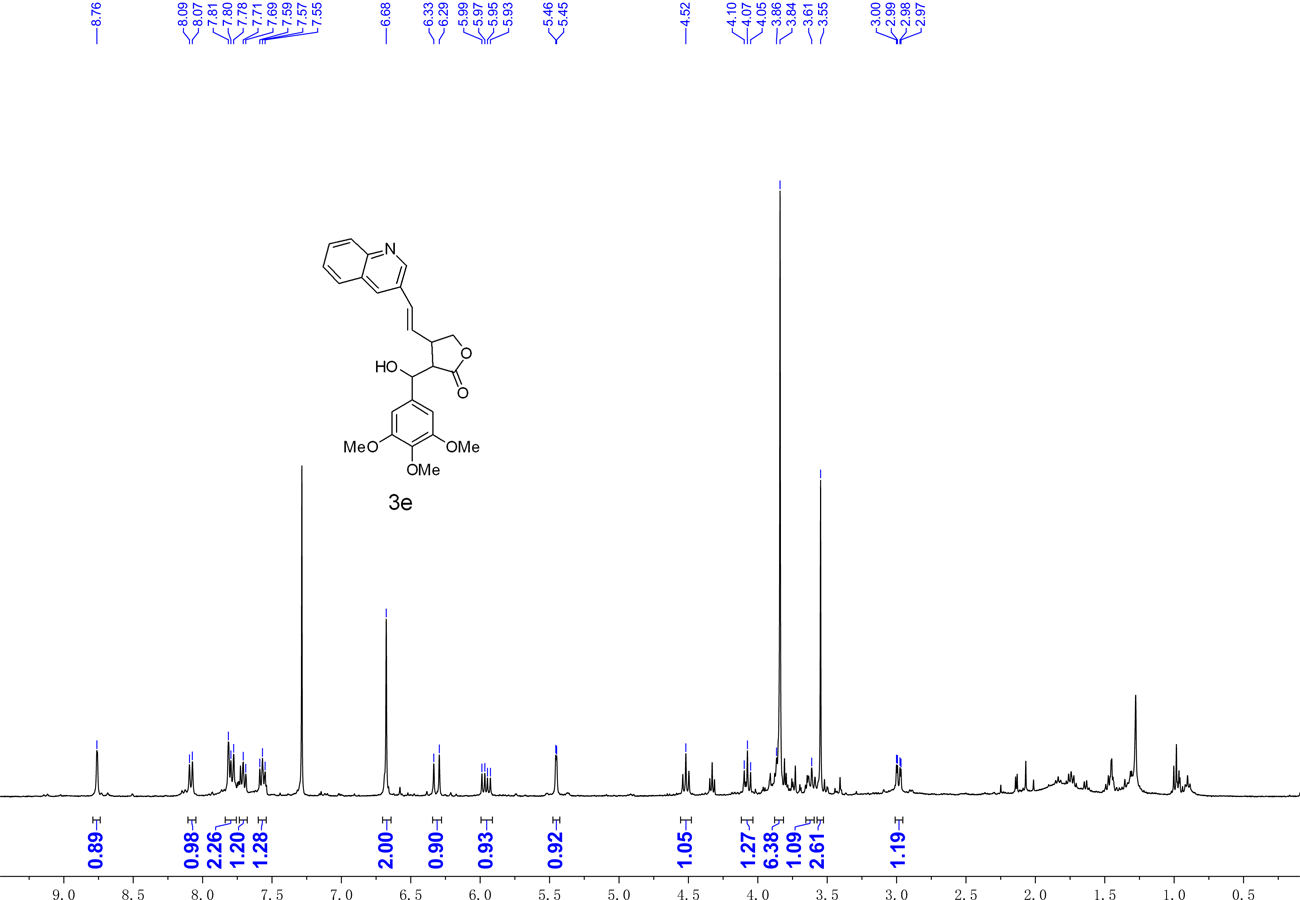


Figure S6 NMR-H spectrum of compound 3f (CDCl3,400MHz)


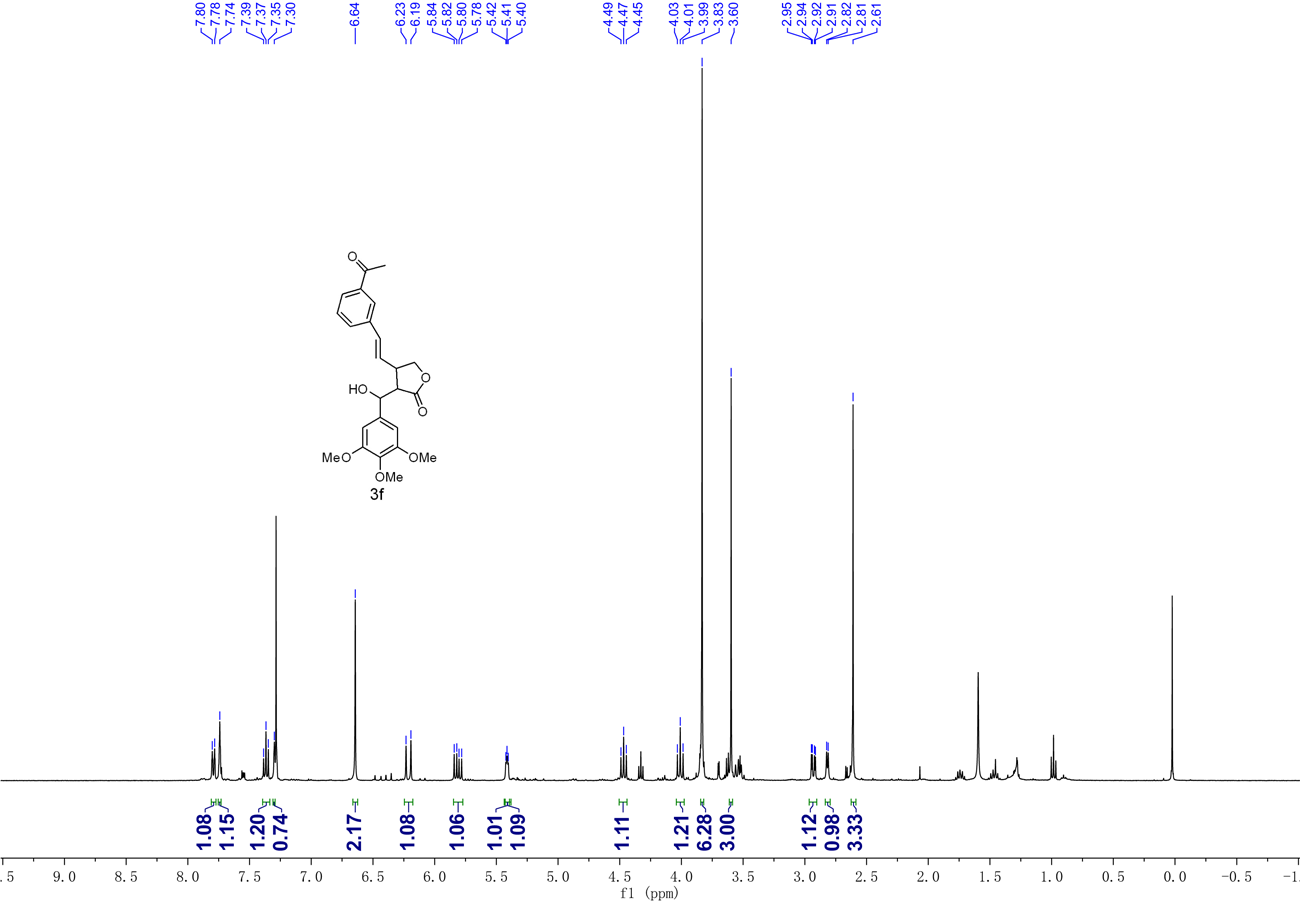


Figure S7 NMR-H spectrum of compound 3g (CDCl3,400MHz)


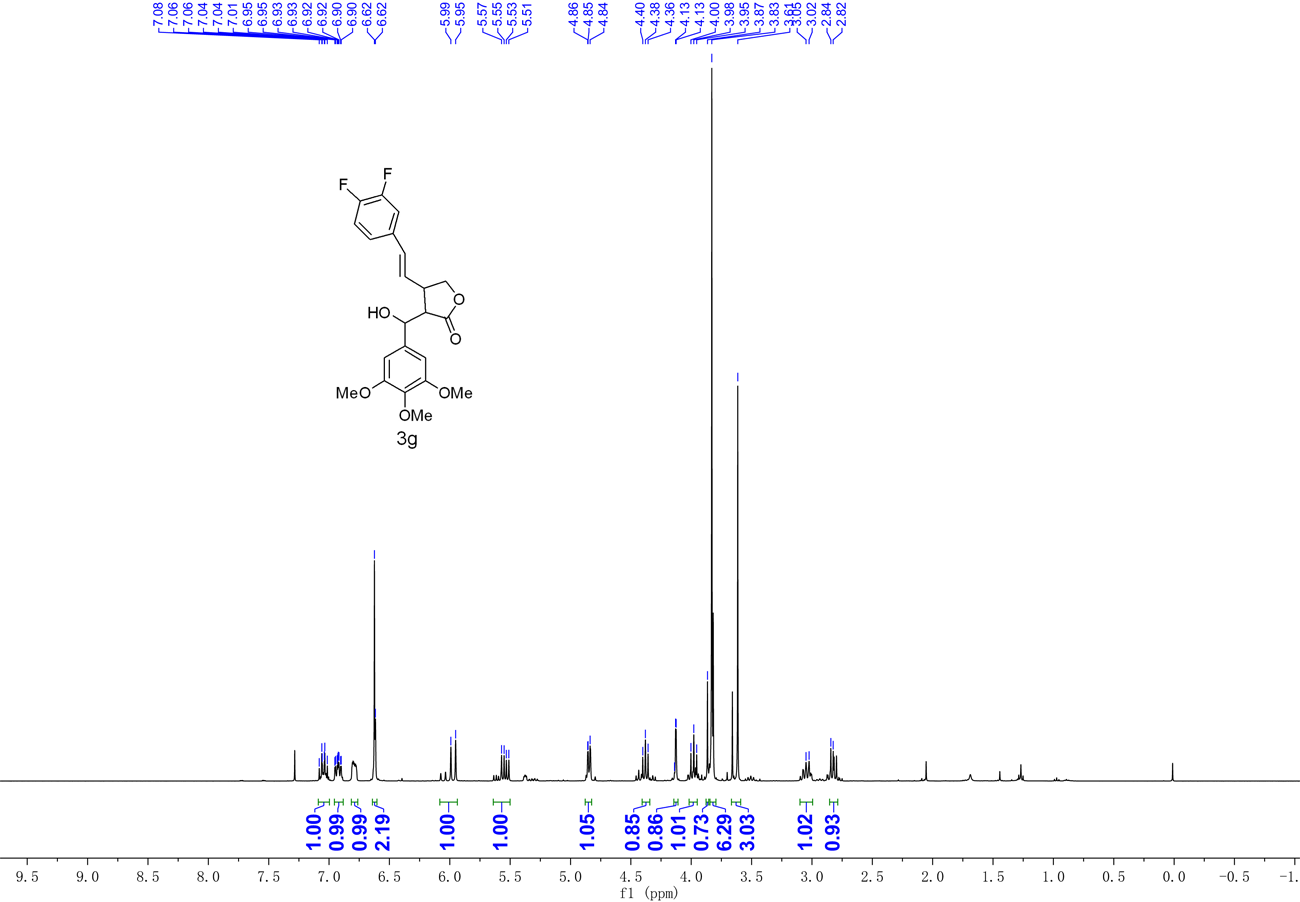


Figure S8 NMR-H spectrum of compound 4 (CDCl3,400MHz)


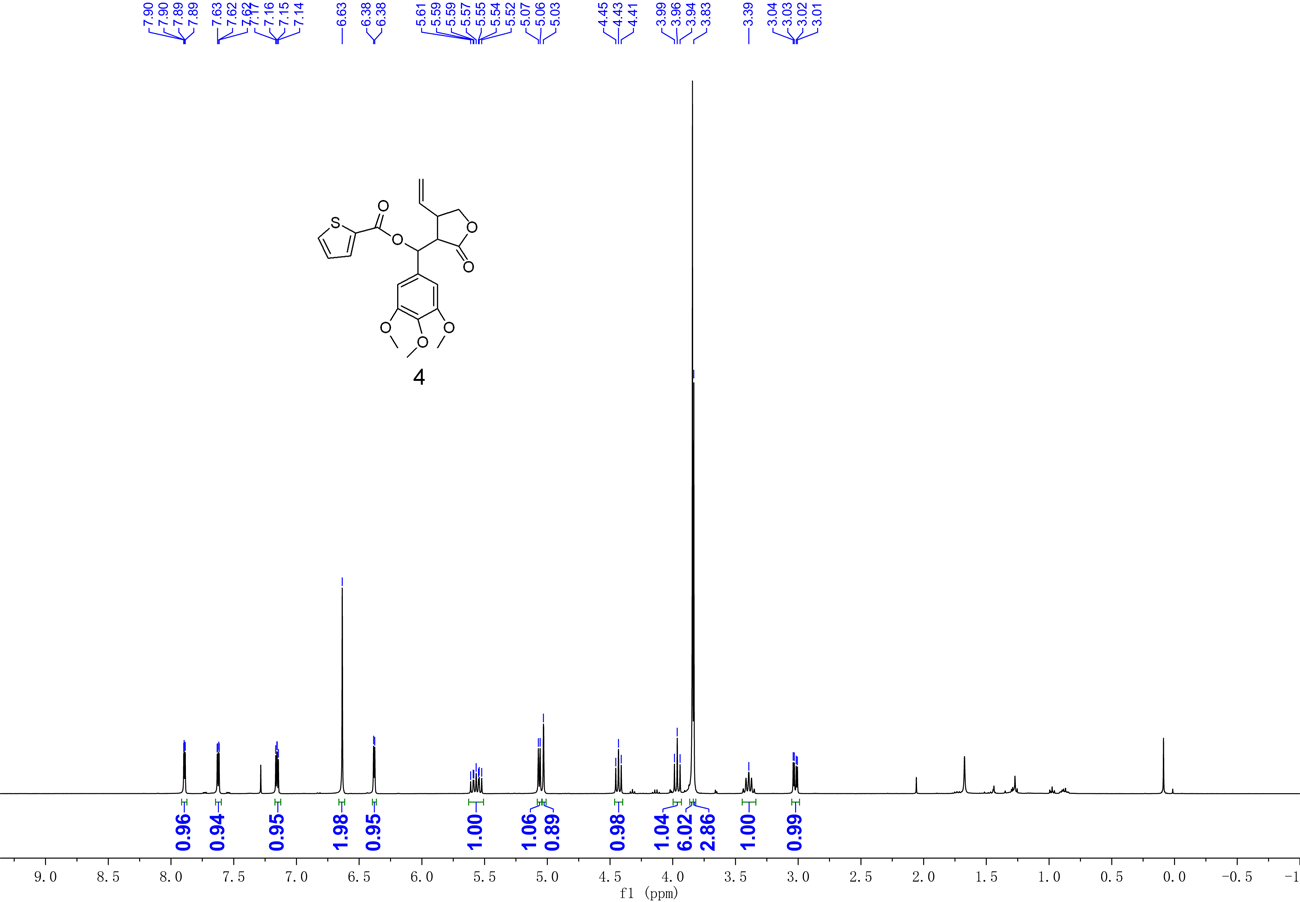

Supplement: Supplementary Information [file srep37323-s1.doc]
